# Daikenchuto attenuates visceral pain and suppresses eosinophil infiltration in inflammatory bowel disease in murine models

**DOI:** 10.1002/jgh3.12410

**Published:** 2020-08-22

**Authors:** Yoko Kogure, Hirosato Kanda, Shenglan Wang, Yongbiao Hao, Junxiang Li, Satoshi Yamamoto, Koichi Noguchi, Yi Dai

**Affiliations:** ^1^ Department of Pharmacy, School of Pharmacy Hyogo University of Health Sciences Kobe Japan; ^2^ Traditional Medicine Research Center Chinese Medicine Confucius Institute at Hyogo College of Medicine (CMCIHCM) Kobe Japan; ^3^ Department of Anatomy and Neuroscience Hyogo College of Medicine Nishinomiya Japan; ^4^ School of Acupuncture‐Moxibustion and Tuina Beijing University of Chinese Medicine (BUCM) Beijing China; ^5^ Division of Gastroenterology, Department of Internal Medicine Dongfang Hospital of BUCM Beijing China

**Keywords:** daikenchuto, eosinophil, inflammatory bowel disease, visceral pain

## Abstract

**Background and Aim:**

Daikenchuto (DKT), a traditional Japanese formula, comprises four herbal medicines and is used for abdominal pain. Inflammatory bowel disease (IBD) includes ulcerative colitis (UC) and Crohn's disease (CD) and is characterized by colonic inflammation and chronic abdominal pain. The present study aimed to investigate whether DKT suppresses colonic hypersensitivity and inflammation associated with IBD in animal models.

**Methods:**

Sprague–Dawley rats were administered 4% sodium dextran sulfate (DSS) or trinitrobenzene sulfate (TNBS) in the colon to establish UC or CD models, respectively. DKT and 5‐aminosalicylic acid (5‐ASA) were administered orally once a day from Days 3 to 7 after induction of colitis. On Day 7, visceral pain and inflammation were evaluated by measuring the visceromotor response (VMR) to colorectal distention (CRD) and inflammatory indicators, including histological score, length of leukocyte infiltration, MPO activity, and eosinophil count.

**Results:**

DSS and TNBS increased VMR to CRD and the inflammation indicators. DKT, but not 5‐ASA, suppressed the VMR to CRD in DSS‐ and TNBS‐treated rats. DKT and 5‐ASA decreased the eosinophil count in both IBD models. In DSS‐treated rats, 5‐ASA, but not DKT, suppressed the MPO activity. In TNBS‐treated rats, neither 5‐ASA nor DKT suppressed MPO activity.

**Conclusion:**

These results suggest that DKT is beneficial for abdominal pain associated with IBD. The anti‐inflammatory effect of DKT on IBD may involve inhibition of eosinophils. The mechanism of anti‐inflammatory effect of DKT partially differs from that of 5‐ASA. Coapplication of DKT and conventional medicine may produce a positive synergy effect for IBD treatment.

## Graphical abstract

To the best of our knowledge, we demonstrated, for the first time, that (i) daikenchuto (DKT) suppressed colonic hypersensitivity and eosinophil infiltration associated with sodium dextran sulfate or trinitrobenzene sulfate‐induced colitis. (ii) The mechanism of anti‐inflammatory effect of DKT partially differs from that of 5‐aminosalicylic acid (5‐ASA). (iii) Coapplication of DKT and 5‐ASA may produce a positive synergy effect for IBD treatment.

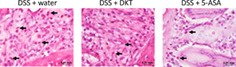

## Introduction

Inflammatory bowel disease (IBD) involves inflammation in the gastrointestinal tract and is accompanied by diarrhea, bloody stool, and chronic abdominal pain. Ulcerative colitis (UC) and Crohn's disease (CD) are two major IBDs, with an increasing number of UC and CD patients every year in the western, as well as developing, countries.^1^, ^2^ Inflammation of the large intestine is frequently observed in UC, and the injured layer of UC is restricted to the mucosa, whereas in CD, inflammation is observed discontinuously throughout the alimentary canal, from mouth to the anus, and the injury reaches the muscular layer. IBD does not cause mortality but adversely affects the quality of life (QOL). 5‐aminosalicylic acid (5‐ASA), steroids, and immunomodulatory agents are preferred medication for IBD treatment. In severe cases, surgery may be required. However, IBD cannot be cured completely, and the present medical treatment strategy provides induction and maintenance for remission. The 5‐ASA remains the mainstay of therapy for induction and maintenance of mild to moderate UC remission.^3^

Daikenchuto (DKT) is a widely used herbal medicine in Japan. It is composed of a combination of four medicines, *Zingiberis rhizoma* (Ginger), *Zanthoxyli fructus* (Japanese pepper), *Panax ginseng* (Ginseng radix), and maltose, and is used for abdominal pain, bloating sensation, or constipation.^4^ In recent years, clinical studies have demonstrated that DKT is effective for preventing ileus after surgical operation, and numerous patients are administered DKT in Japan.^5^, ^6^ Several studies have reported that DKT enhances the gastrointestinal motility and promotes gastric blood flow and has an anti‐inflammatory effect.^5^, ^7^, ^8^ Considering the increasing practice of DKT administration in gastrointestinal disease, we investigated whether DKT can be used in IBD for analgesic and anti‐inflammatory effects.

In the present study, we assessed the effect of DKT in suppressing abdominal pain or colonic inflammation in UC and CD animal models and tried to elucidate the mechanism by which DKT exerted analgesic and anti‐inflammatory effect on IBD animals by comparing it with the existing medicine 5‐ASA.

## Methods

### *Animals*

All animal experimental procedures were approved by the Hyogo University of Health Sciences Committee on Animal Research (No. 2011‐19, 2012‐24, 2014‐02, 2016‐08) and were performed in accordance with the National Institutes of Health guidelines on animal care. Adult male Sprague–Dawley rats (190–210 g) (Japan SLC Inc., Shizuoka, Japan) were used in the study. Animals were housed in plastic cages in groups of two or three at a constant temperature of 22 ± 2 °C and 60–80% humidity with free access to food and water under a 12‐h light/dark cycle. The treatments were carried out randomly in groups.

### *Colitis models*

To induce visceral pain and chronic inflammation in the colon, we created two types of colitis models: DSS‐induced colitis as UC and TNBS‐induced colitis as CD. For DSS‐induced colitis, rats had free access to 4% DSS (M.W. 36 000–50 000) drinking water for 7 days, whereas control rats had access to distilled water. For TNBS‐induced colitis, rats were lightly anesthetized with 2% isoflurane (Wako, Osaka, Japan), and a feeding needle (Fuchigami, Kyoto, Japan) was inserted 5 cm from the anus to administer TNBS in colon (7.5 mg/rat, dissolved in 0.2 mL saline with 50% ethanol), whereas control rats received 0.2 mL saline. All rats were maintained for 1 h under 1.5% isoflurane after TNBS infusion. The colitis induction was carried out on Day 0, and the rats were used for experiments on Day 7. Body weight, intestine length, and intestine weight were measured on Day 7. Rats that lost more than 7% body weight after TNBS treatment were excluded from the experiments.

### *Visceromotor response to colorectal distention*

For assessing the visceral pain, the visceromotor response (VMR) to colorectal distention (CRD) was measured using electromyography (EMG), as described previously.^9^ Briefly, under isoflurane anesthesia, a latex balloon (Okamoto, Tokyo, Japan) was inserted into the distal colon, and needle electrodes (AD instruments, Bella Vista, Australia) were placed on the right external oblique musculature for obtaining EMG recordings. Next, rats were anesthetized with 0.5% propofol (5 mL/h for 5 min and 6–7.5 mL/h/kg continuous intravenous administration), and their EMG signals were recorded after recovery from isoflurane anesthesia (approximately 30 min) using a data acquisition system equipped with a software (PowerLab 4/25, Chart, AD instruments). After evaluating the visceral pain, CRD was performed thrice by tonic inflation up to 60 mmHg over a period of 10 s with an interval of 5 min. VMR to CRD was quantified as the maximal value of area under the curve (AUC) of EMG for 5 s.

### *Histology*

Rats were sacrificed on Day 7 after measurement of VMR to CRD. A total of 5 cm of rectum/colon from the anus was resected from DSS‐ or TNBS‐treated or control rats. The inflamed region (0.5–1 cm) of the colon or its equivalent in saline‐treated rats was subjected to staining. The excised colorectal tissue was fixed in 4% paraformaldehyde for 3 nights and was then dehydrated in 20% sucrose for 3 days at 4 °C. The colorectal tissues were sliced into 10‐μm sections and stained with HE. We evaluated the histological damage according to three indices: histological score, length of leukocyte infiltration, and eosinophil count. The histological score was assessed as the depth of the ulcers and infiltration of inflammatory cells on a scale of 0–3 (Table 1), as previously described.^10^ The histological score and length of leukocyte infiltration were observed at 40‐fold magnification, and the number of eosinophils was counted at 400‐fold magnification.

**Table 1:** Histological score

Score	Depth of the ulcers	Infiltration of inflammatory cells
0	No ulceration	None
1	Erosion	Focal infiltration of the mucosa
2	Ulceration to the submucosa	Focal infiltration of the mucosa to submucosa (the submucosal infiltration area within quarter length)
3	Ulceration over the submucosa	Severe infiltration except for the aforementioned

Total score of depth of the ulcers and infiltration of inflammatory cells was assessed as histological score.

### *MPO* *assay*

Rats were sacrificed on Day 7; subsequently, 5 cm of the intestine from the anus was resected from DSS‐ or TNBS‐treated or control rats. The inflamed region (0.5–1 cm) of the removed intestine was assayed. The excised colon was cut into 1‐mm squares, homogenized in 1 mL of 50 mM potassium phosphate buffer (pH 6.0) containing 0.5% hexadecyl trimethyl ammonium bromide (HTAB) (Sigma‐Aldrich Co., St. Louis, MO, USA), and centrifuged at 15 000 rpm at 4 °C for 30 min. The supernatant was used for the MPO assay. The sample was mixed in a ratio of 1:30 with 0.2 M phosphate buffer (pH 6.0) containing 0.167 mg/mL *o*‐dianisidine dihydrochloride (Tokyo Chemical Industry Co. Ltd., Tokyo, Japan) and 0.0005% H_2_O_2_, and the absorbance was measured after 15 min of incubation at 460 nm.

### *Drug administration*

Rats were orally administered with one of the following medicines: DKT (75 mg/kg), 5‐ASA (100 mg/kg), or tap water (5 mL/kg per administration). The dose of DKT used in this study has been wildly applicated for animal studies.^8^, ^11^ Drugs were administered once a day from Days 3 to 7 (5 consecutive days), and on Day 7, the drugs were administered >2 h before the experiment.

### *Chemicals*

DSS (MP Biomedicals, Santa Ana, CA, USA) was dissolved in distilled water. Propofol (Maruishi Pharmaceutical Co. Ltd., Osaka, Japan) was diluted to 0.5% w/v with saline; 0.5% hematoxylin aqueous solution and 0.5% eosin aqueous solution were purchased from Merck‐Millipore (Darmstadt, Germany). DKT powder (without maltose) was provided by Tsumura & Co. (Tokyo, Japan) and was dissolved in tap water. To prepare 5‐ASA, Pentasa (KYORIN Pharmaceutical Co. Ltd., Tokyo, Japan) was crushed and dissolved in tap water. Drugs other than those previously mentioned were purchased from Nacalai Tesque (Kyoto, Japan).

### *Statistical analysis*

Data were expressed as mean ± SEM. *t*‐test or one‐way ANOVA followed by post hoc Dunnett test as parametric test and Mann–Whitney *U* test as nonparametric test were performed to evaluate all data using Statcel3 for Excel (OMS Publishing Inc., Saitama, Japan). A value of *P* < 0.05 was considered a statistically significant difference.

## Results

### *DKT* *attenuated* *DSS‐* *or* *TNBS‐induced visceral hypersensitivity*

**Figure 1:**
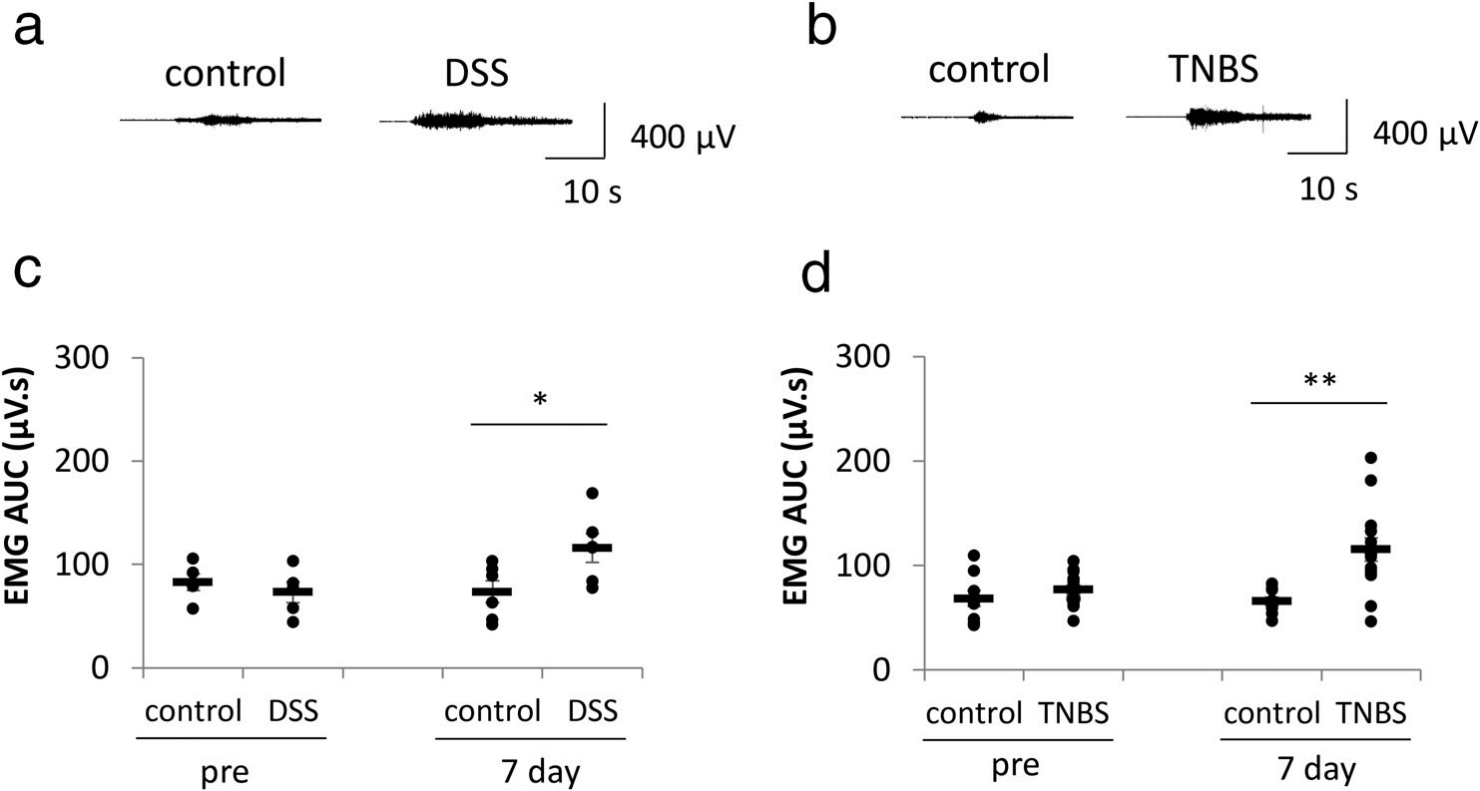
Sodium dextran sulfate (DSS) or trinitrobenzene sulfate (TNBS) treatment induced colonic hypersensitivity. Representative traces of electromyography (EMG) recordings for DSS group (a) and TNBS group (b). Averaged area under the curve (AUC) values of EMG before (pre) and 7 days after (7 day) drug treatment in DSS (c) and TNBS (d). DSS‐ or TNBS‐treated rats were more sensitive to colorectal distention at 60 mmHg than the control rats. The horizontal bars indicate average values. Data are expressed as the mean ± SEM (*P < 0.05, **P < 0.01; *vs* control, *t*‐test, *n* = 5–14 in each group).

**Figure 2:**
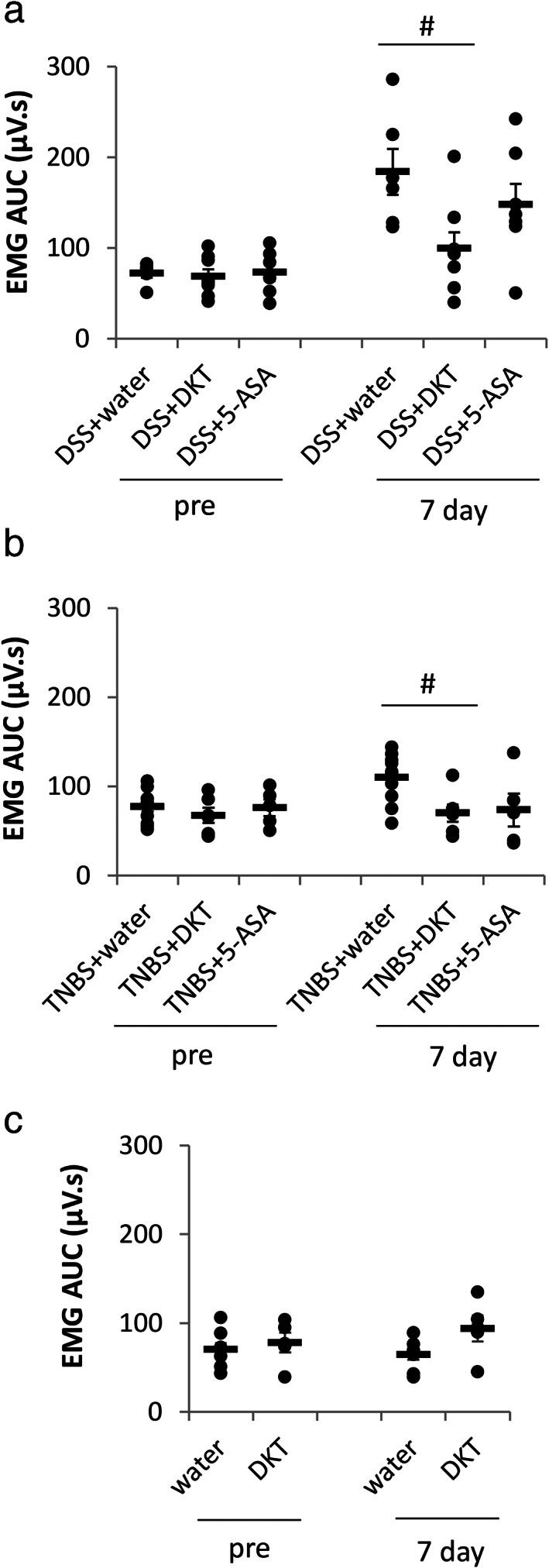
Daikenchuto (DKT) attenuated sodium dextran sulfate (DSS)‐ and trinitrobenzene sulfate (TNBS)‐induced colonic hypersensitivity (a, b). DKT had no effect on visceromotor response to colorectal distention of control group (c). The horizontal bars indicate average values. Data are expressed as the mean ± SEM (#*P* < 0.05; *vs* DSS or TNBS + water, one‐way ANOVA, *n* = 5–11 in each group).

We established two different IBD models and tested their visceral sensitivity to colonic distension. We found that VMR to CRD at 60 mmHg distension was significantly increased in 7 days after DSS (Fig. 1a,c) or TNBS (Fig. 1b,d) treatment compared to their respective controls, indicating that these rats developed visceral hyperalgesia. These data are in accordance with the previous reports.^12^, ^13^, ^14^ To investigate whether DKT had an analgesic effect on DSS‐ or TNBS‐treated rats, the rats were administered DKT orally for 5 days from Days 3 to 7 after DSS or TNBS treatment. In the control group, the rats were administered 5‐ASA. We found that DKT but not 5‐ASA significantly suppressed the VMR to CRD in both DSS‐ and TNBS‐treated rats (Fig. 2a,b, respectively). VMR to CRD of control rats were unaffected by DKT (Fig. 2c).

### *IBD* *rats presented colorectal tissue inflammation with eosinophil infiltration*

**Figure 3:**
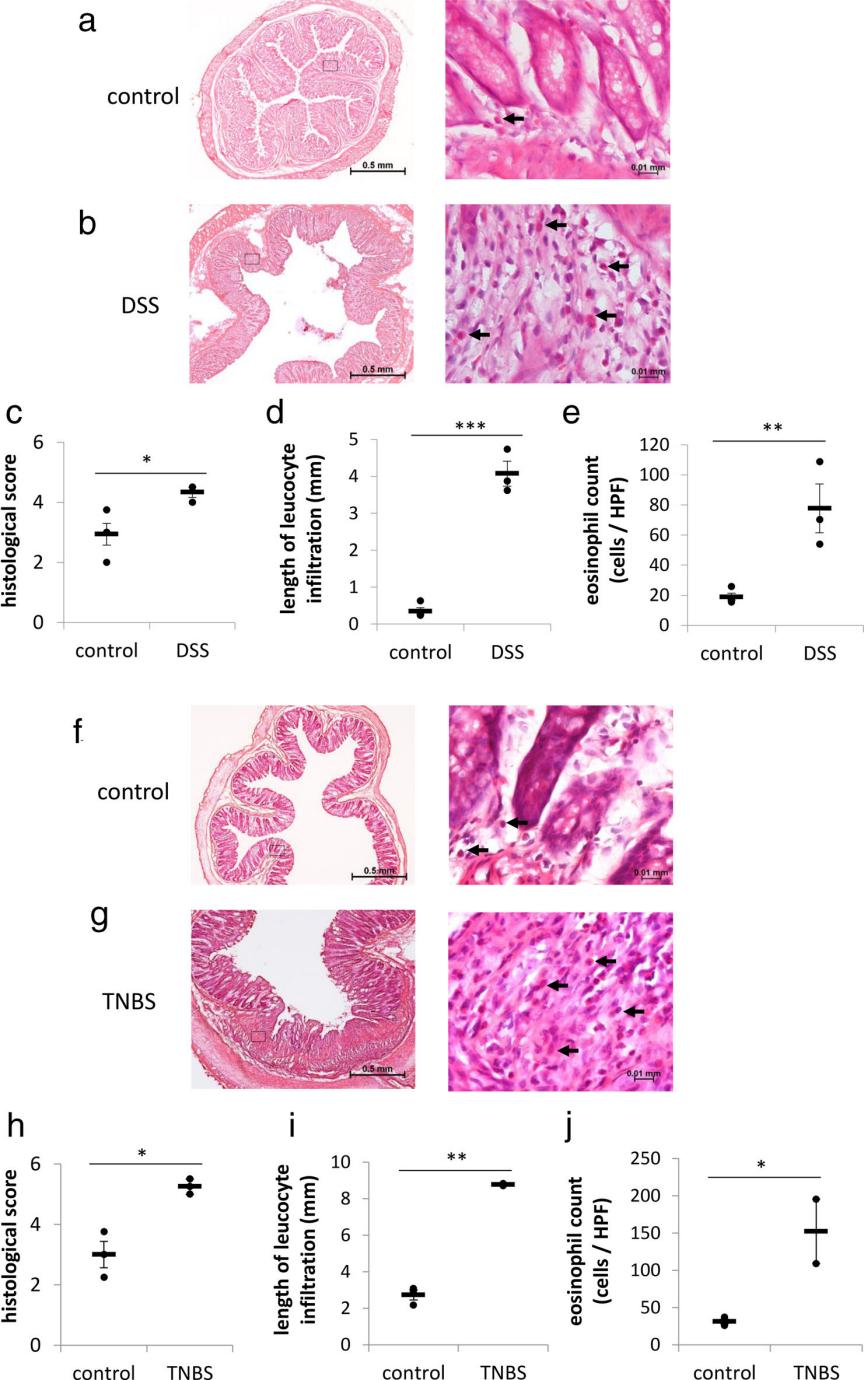
Sodium dextran sulfate (DSS) or trinitrobenzene sulfate (TNBS) treatment induced inflammatory cell infiltration in the colonic tissue. Representative HE‐stained images of colon on Day 7 in control (a) and DSS (b) groups at low‐power field (LPF) and high‐power field (HPF). Bar graph presents individual histological score (c), length of leucocyte infiltration at LPF (d), and number of inflammatory cells at HPF (e) in DSS‐treated rats on Day 7. Representative HE‐stained images of colon on Day 7 in control (f) and TNBS (g) group at LPF and HPF. Bar graph depicts the individual histological score (h), length of leucocyte infiltration at LPF (i), and number of inflammatory cells at HPF (j) in the most severely damaged area of colon in TNBS‐treated rats. Arrows in the HE‐stained images indicate eosinophils. The horizontal bars indicate average values. Data are expressed as the mean ± SEM (*P < 0.05, **P < 0.01, ***P < 0.001; *vs* control, *t*‐test, *n* = 3–4 in each group).

We performed HE staining of the colorectal tissues and assessed their inflammation grade by evaluating the histological score and the length of leucocyte infiltration. Colon tissues revealed clear columnar epithelium and crypts with few leucocyte staining in mucosa in the normal rats (Fig. 3a,f), whereas the disrupted epithelium–crypts architecture and severe leucocyte infiltration was observed in DSS‐ or TNBS‐treated rats (Fig. 3b,g). The histological score and length of leucocyte infiltration in DSS‐ and TNBS‐treated rats were significantly higher than those in respective control rats (Fig. 3c,d,h,i). As HE staining can clearly identify eosinophils, we counted the number of eosinophils in each isolated colorectal tissue. The eosinophil count in DSS‐ and TNBS‐treated rats was significantly increased compared to those in the respective control rats (Fig. 3e,j). Considering the aforementioned inflammatory indices, the severity of tissue damage in TNBS‐treated rats was higher than that in DSS‐treated rats.

### *DKT* *suppressed excess eosinophils but not leucocyte infiltration and* *MPO* *activity in* *IBD* *rats*

**Figure 4:**
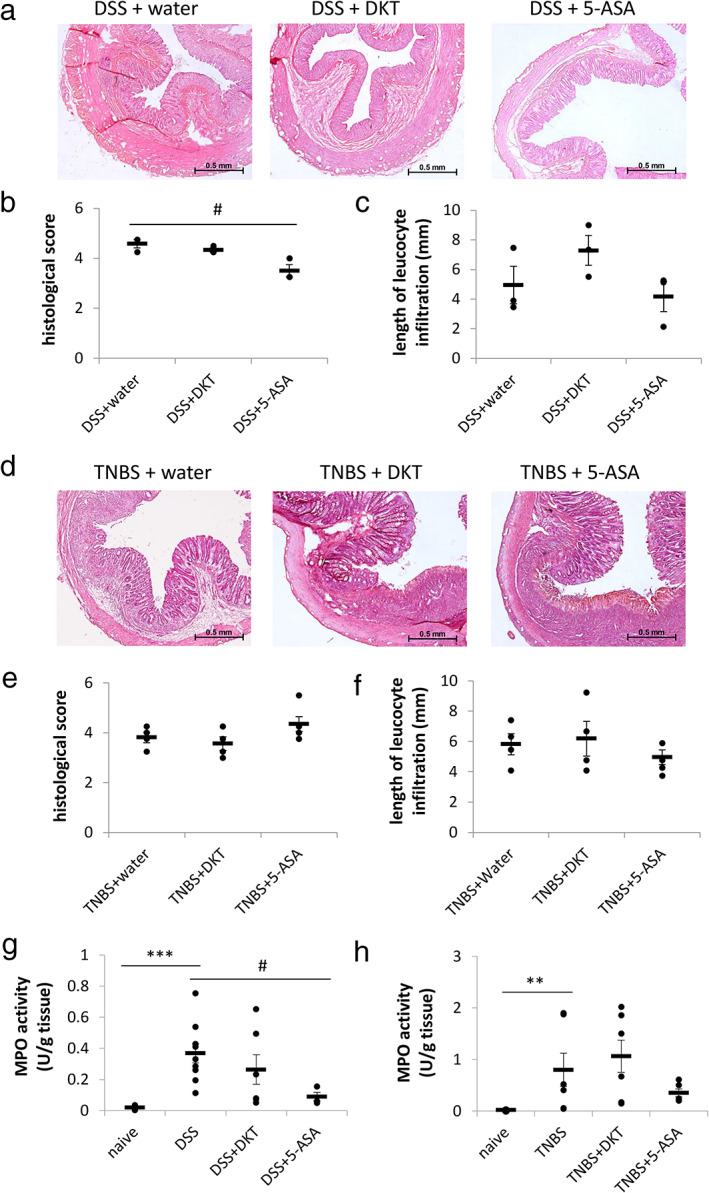
Daikenchuto (DKT) did not suppress the inflammatory scores in inflammatory bowel disease rats. Representative HE‐stained images of colon at low‐power field (LPF) with sodium dextran sulfate (DSS) (a) or trinitrobenzene sulfate (TNBS) (d) administration. Histological score in DSS (b) and TNBS (e) groups. Individual length of leucocyte infiltration at LPF in DSS (c) and TNBS (f) groups. DSS‐ (g) and TNBS‐treated (h) rats increased the MPO activity. The horizontal bars indicate average values. Data are expressed as the mean ± SEM (**P < 0.01, ***P < 0.001; *vs* naïve, *t*‐test, #*P* < 0.05; *vs* DSS or DSS + water, *t*‐test or *U*‐test, *n* = 3–9 in each group).

**Figure 5:**
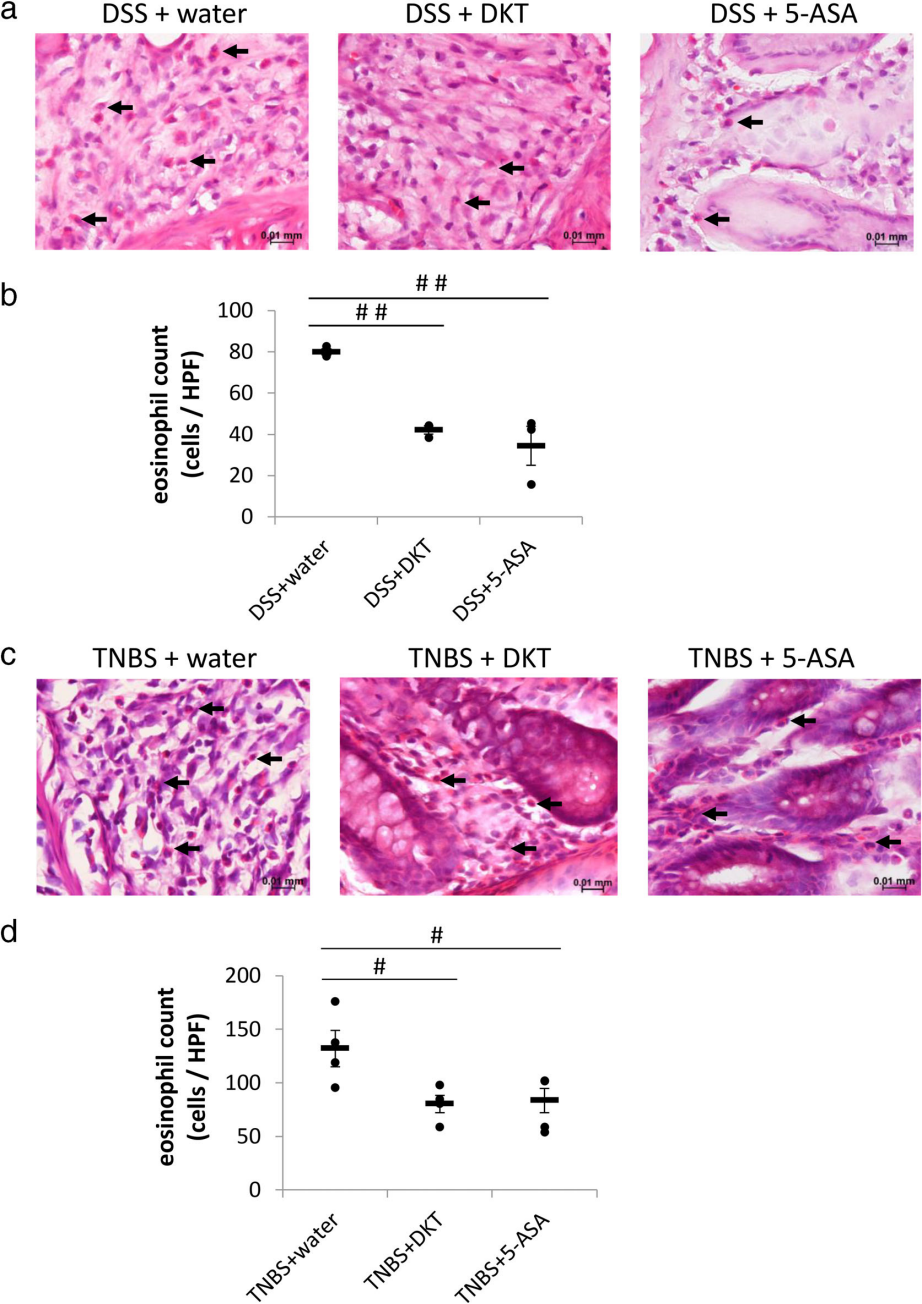
Daikenchuto (DKT) suppressed the eosinophils in colonic tissue induced by sodium dextran sulfate (DSS) and trinitrobenzene sulfate (TNBS). Representative HE‐stained images of colon at high‐power field (HPF) with DSS (a) or TNBS (c) administration. Average eosinophil count at HPF in the colon after drug treatment in DSS (b) and TNBS (d). The horizontal bars indicate average values. Data are expressed as the mean ± SEM (#*P* < 0.05, ##*P* < 0.01; *vs* DSS or TNBS + water, one‐way ANOVA, *n* = 3–5 in each group).

As DKT is reported to have an anti‐inflammatory effect,^8^, ^15^ we investigated whether it could suppress colorectal inflammation in the IBD rats. Unexpectedly, DKT failed to improve the histological score and length of leucocyte infiltration in both DSS‐ and TNBS‐treated rats (Fig. 4a–f). Furthermore, to assess the effect of DKT on neutrophils, we evaluated MPO activity in IBD rats after DKT treatment. We observed that MPO activity was significantly increased in both DSS‐ and TNBS‐treated rats; however, DKT did not suppress these activities (Fig. 4g,h). We also confirmed body weight, length, and weight of intestine on Day 7 (Table 2). DSS treatment significantly decreased the body weight, shortened the intestine length, and increased the intestine weight. DKT and 5‐ASA suppressed the shortening and increasing of the intestine weight in DSS‐treated rats. Nevertheless, the eosinophil count in colorectal tissues in both DSS and TNBS rats was significantly attenuated by DKT treatment (Fig. 5). Treatment of 5‐ASA significantly suppressed the increasing histological score and MPO activity in DSS‐treated rats but not in TNBS‐treated rats. Moreover, MPO activity had a strong inhibitory effect in 5‐ASA‐treated rats (Fig. 4g,h). 5‐ASA also reduced the eosinophil count in both DSS and TNBS rats (Fig. 5).

**Table 2:** Body weight, intestine length, and weight on Day 7

	Naive	DSS	DSS + DKT	DSS + 5‐ASA	TNBS	TNBS + DKT	TNBS +5‐ASA
Body weight (% of 0 day)	126.5 ± 1.2	118.5 ± 0.6***	115.5 ± 1.3***	118.8 ± 2.5**	126.5 ± 1.6	126.3 ± 1.6	127.4 ± 1.1
Intestine length (cm)	18.5 ± 0.4	16.4 ± 0.4**	17.4 ± 0.6	16.7 ± 0.9	18.7 ± 0.5	18.6 ± 0.7	19.5 ± 0.5
Intestine weight (mg/cm)	65.2 ± 0.9	77.0 ± 3.5**	74.0 ± 6.5	71.0 ± 4.6	63.9 ± 3.1	60.8 ± 3.2	59.4 ± 3.0

Data are expressed as the mean ± SEM (**P < 0.01, *** *P* < 0.001; *vs* naive, *t*‐test, *n* = 3–9 in each group).

5‐ASA, 5‐aminosalicylic acid; DKT, daikenchuto; DSS, sodium dextran sulfate; TNBS, trinitrobenzene sulfate.

## Discussion

In the present study, we investigated the analgesic and anti‐inflammatory effects of DKT on IBD rats. This is the first study to demonstrate that administration of DKT for 5 days could attenuate colonic hypersensitivity and reduce eosinophil infiltration in both DSS‐ and TNBS‐induced IBD rats. In contrast, the existing IBD medicine, 5‐ASA, did not suppress DSS‐ or TNBS‐induced colonic hypersensitivity; however, it reduced the inflammatory indices, including MPO activity and eosinophil infiltration, in DSS rats. Although both DKT and 5‐ASA decreased the eosinophil count, only DKT revealed an analgesic effect on visceral pain accompanied with the IBD models. This indicated that eosinophils seem to be unrelated to the analgesic mechanism of DKT. Therefore, the mechanism underlining DKT's analgesic effect on IBD rats may be independent of the bowel inflammatory condition.

The analgesic effect of DKT on IBD rats may be a result of antinociceptive action of [6]‐gingerol, a major compound in ginger, which is one of the DKT components. [6]‐gingerol has been reported to reduce acetic acid‐induced writhing response, decrease formalin‐induced licking time in the late phase, and has an antiallodynic effect on spinal nerve ligation model rats.^16^, ^17^, ^18^ Moreover, previous studies indicated that gingerol and shogaol (another major active ingredients of ginger) and hydroxy‐α‐sanshool (a major active ingredient of Japanese pepper) could stimulate and desensitize two pain sensors, the transient potential receptor vanilloid 1 (TRPV1) and transient potential receptor ankyrin 1 (TRPA1) channels. Therefore, ginger and Japanese pepper may play an essential role in the analgesic effect of DKT. We did not find the inhibitory effect of DKT on VMR to CRD in naïve rats (Fig. 2c), which is contradictory to a previous study.^19^ The discrepancy may be attributed to a different experimental protocol for DKT administration, wherein DKT was administered only once, and the VMR to CRD was measured in conscious rats; in contrast, we administered DKT for 5 days and assessed the VMR under anesthesia. Anesthesia in the present study may mask the analgesic effect of DKT in naïve rats.

MPO is a peroxidase enzyme that is abundantly expressed in neutrophils. MPO activity is proportional to the neutrophil count.^20^ In this study, we confirmed that MPO activity significantly increased in both DSS‐ and TNBS‐treated rats, which is in accordance with the previous reports.^10^, ^21^, ^22^ We also found that an abundance of eosinophils accumulated into the inflammatory colorectal mucosa in the IBD rats, which is in accordance with other studies using both animal models and patients.^23^, ^24^, ^25^, ^26^, ^27^ These results indicated that IBD involved an increase in both neutrophils and eosinophils in the local tissues. Clinical studies have reported that the accumulation of both neutrophils and eosinophils is closely related to IBD.^28^, ^29^

Interestingly, DKT selectively suppressed the eosinophils but not neutrophils. Neutrophils are the most abundant leukocytes that normally reside in blood but are absent in the intestinal mucosa. An excessive recruitment of neutrophils from the blood circulation to the inflamed local colorectal mucosa occurs in IBD, leading to crypt abscess formation and damage of mucosal architecture.^30^ In contrast, eosinophils are primarily located in the normal intestinal mucosa rather than in the blood; the resident intestinal eosinophils have multiple functions, such as maintenance of mucosal barrier, provision of immunity to pathogens, interactions with the enteric nervous system, and linking innate and adaptive immunity.^31^ In case of IBD, eosinophils accumulate in addition to the residential eosinophils in inflamed mucosa and result in a massive release of both cytotoxic granule proteins and proinflammatory cytokines.^31^ Considering 5‐ASA, but not DKT, suppressed MPO (neutrophils function) in DSS rats (Fig. 4g), the mechanism underlying the anti‐inflammatory effect of DKT partly varies from that of 5‐ASA.

In addition, eosinophils in IBD may play pivotal roles apart from the inflammatory response, for example, recent studies have demonstrated that eosinophils might be associated with tissue remodeling/repair or fibrosis in IBD rats.^32^, ^33^, ^34^ Remodeling events were found to be accompanied by a massive eosinophil infiltrate and widespread eosinophil degranulation.^35^ DKT promotes intestinal epithelial wound healing in IBD rats,^36^ which might be a result of the attenuating effect of DKT on the eosinophil accumulation, as observed in the present study.

Previous studies have reported that intestinal eosinophils can produce transforming growth factor (TGF)‐β to facilitate fibrosis by activating myofibroblast cells.^37^ Fibrosis occurred in IBD patients, particularly in CD (about 30%).^33^ TNBS also induced fibrosis in animals.^33^ Recent reports indicated that DKT could ameliorate fibrosis by suppressing the downstream process of TGF‐β1 receptor signaling pathway in myofibroblasts.^38^, ^39^ Our data demonstrating that DKT suppressed eosinophils in IBD rats provide a rational link between the DKT, eosinophils, and fibrosis. Eosinophil reduction by DKT may suppress the production of TGF‐β and subsequently prevent fibrosis in colitis. Ginger and Japanese pepper are related to the suppression of TGF‐β.^39^ Ginsenoside Rb1 and its metabolite compound K can inhibit the inflammatory response by suppressing NF‐κB and interleukin‐1 receptor‐associated kinase 1 activation.^40^, ^41^ These medicines in DKT may collaboratively suppress the inflammatory response initiated by eosinophil increase.

Considering that DKT suppressed eosinophils infiltration, it can be expected to be used not only for IBD but also for eosinophilic gastrointestinal disorder, such as eosinophilic esophagitis and eosinophilic gastroenteritis.

In summary, our study demonstrated that DKT suppressed the colonic hypersensitivity, as well as eosinophil infiltration associated with DSS‐ or TNBS‐induced colitis. In contrast, 5‐ASA suppressed both neutrophil and eosinophil but had no analgesic effect, indicating that the anti‐inflammatory mechanism of DKT partially differs from that of 5‐ASA. Therefore, DKT is beneficial for abdominal pain associated with IBD, and coapplication of DKT and 5‐ASA may produce a positive synergy effect for IBD treatment. Detailed investigation is required to clarify the mechanism by which DKT attenuates pain and the type of herbal medicine mainly involved in the process.
